# Differential Rotational Movement of the Thoracolumbosacral Spine in High-Level Dressage Horses Ridden in a Straight Line, in Sitting Trot and Seated Canter Compared to In-Hand Trot

**DOI:** 10.3390/ani11030888

**Published:** 2021-03-20

**Authors:** Russell MacKechnie-Guire, Thilo Pfau

**Affiliations:** 1 Centaur Biomechanics, 25 Oaktree Close, Moreton Morrell, Warwickshire CV35 9BB, UK; 2 Department of Clinical Science and Services, The Royal Veterinary College, Hawkshead Lane, Brookman’s Park, Hatfield AL9 7TA, UK; tpfau@rvc.ac.uk

**Keywords:** sensors, IMUs, markers, pitch, roll and heading, locomotion, skin

## Abstract

**Simple Summary:**

As part of the investigative process of “loss of athletic performance”, quantitative data may help veterinary decision making when assessing equine back dysfunction. Ranges of motion of differential rotational movement were quantified between adjacent inertial measurement units, which were attached to the skin over the thoraco–lumbo–sacral area in 10 dressage horses. Differential rotational movements were collected during trot in-hand and ridden in sitting trot/canter during straight-line locomotion. For the thoracic area, differential heading values were smaller in sitting trot and canter compared to trotting in-hand. Compared to trotting in-hand the thoraco-lumbar differential pitch values were higher in sitting trot and canter. The lumbo-sacral region, differential pitch values were increased in canter compared to trotting in-hand and differential heading values were higher in sitting trot compared to canter. Compared to in-hand, reduced heading values were measured in the cranial–thoracic area and increased in the caudal–thoracic and lumbar area. Pitch values increased with ridden exercise from the caudal–thoracic to the sacral area. Back movement alters when ridden compared to an unloaded condition. Understanding back movement whilst being ridden will help advance our understanding on equine back movement and assist with decision making.

**Abstract:**

Assessing back dysfunction is a key part of the investigative process of “loss of athletic performance” in the horse and quantitative data may help veterinary decision making. Ranges of motion of differential translational and rotational movement between adjacent inertial measurement units attached to the skin over thoracic vertebrae 5, 13 and 18 (T5, T13, T18) lumbar vertebra 3 (L3) and tuber sacrale (TS) were measured in 10 dressage horses during trot in-hand and ridden in sitting trot/canter. Straight-line motion cycles were analysed using a general linear model (random factor: horse; fixed factor: exercise condition; Bonferroni post hoc correction: *p* < 0.05). At T5-T13 the differential heading was smaller in sitting trot (*p* ≤ 0.0001, 5.1° (0.2)) and canter (*p* ≤ 0.0001, 3.2° (0.2)) compared to trotting in-hand (7.4° (0.4)). Compared to trotting in-hand (3.4° (0.4)) at T18-L3 differential pitch was higher in sitting trot (*p* ≤ 0.0001, 7.5° (0.3)) and canter (*p* ≤ 0.0001, 6.3° (0.3)). At L3-TS, differential pitch was increased in canter (6.5° (0.5)) compared to trotting in-hand (*p* = 0.006, 4.9° (0.6)) and differential heading was higher in sitting trot (4° (0.2)) compared to canter (*p* = 0.02, 2.9° (0.3)). Compared to in-hand, reduced heading was measured in the cranial–thoracic area and increased in the caudal–thoracic and lumbar area. Pitch increased with ridden exercise from the caudal–thoracic to the sacral area.

## 1. Introduction

Loss of athletic performance in the horse can be attributed to multiple factors, which can be investigated using various diagnostic techniques. Back dysfunction is a key part of the investigative process of “loss of athletic performance” in the horse [1]. Diagnosing the causes of back related dysfunction remains a diagnostic and therapeutic challenge in relation to the multifactorial aetiopathogenesis [2,3,4]. Across gaits and exercises, the vertebral column shows different movements, which can be decomposed into three rotational movements, categorized into flexion–extension, lateral bending and axial rotation, and three translational movements, categorized into dorsoventral, mediolateral and craniocaudal translation [5,6,7,8]. Initial in vitro studies quantified the extent to which the different parts of the spinal column were able to move [1,9,10,11,12]. This was followed up with studies making use of treadmill locomotion and with the insertion of Steinman pins into the tips of the dorsal spinous processes, reporting differential vertebral kinematics in vivo for the thoracic, lumbar, and sacral regions showing the difference in amplitudes and the distribution between the different rotational components in walk [8], trot [6] and canter [7].

The rotational movement of the thoraco–lumbo–sacral area was also measured with 3D optical motion capture making use of external, skin-mounted markers as a non-invasive approach [13]. However, skin displacement is a well-recognised limitation with motion capture, with increasing displacement in the proximal regions (distal scapula and greater trochanter) when walking [14] and trotting [15]. The relative motion of the skin can be distorted in relation to the underlying bony structures due to the non-rigid attachment of the skin to the bony structure. Specifically for back movement, there is also a discrepancy between the centre of rotation of the vertebral body and the position of the skin-fixated marker over the dorsal spinous process [16]. Regarding thoraco–lumbo–sacral movement in walk and trot with skin-mounted markers have been reported to determine flexion–extension of the thoracolumbar spine and axial rotation of the sacrum satisfactorily. In walk, lateral bending values were reported to be accurate for the vertebral segments of the mid thoracic and the cranial lumbar vertebra, whereas in trot valid data were reported for the thoracolumbar spine [16].

The presence of a rider can influence lameness scores [17], as well as variability of velocity and acceleration in a forward direction [18] and can lead to systematic changes in movement symmetry [19,20]. The horse’s back movement also alters with added weight, increasing the overall extension of the lumbar spine in walk and trot with a saddle with a dead weight (75 kg) [21]. Different riding positions also affect the movement of the back, with the addition of a rider leading to an overall extending effect on the back in sitting trot [22]. In rising trot, maximal flexion appears to be similar to the unloaded condition, whilst maximal extension was similar to sitting trot and lateral bending of the lumbar spine increased [22]. These changes in back movement, as a result of both a static mass and the dynamic weight of a rider, emphasise the relevance of a ridden component as part of a “loss of athletic performance assessment” as a way of visually (or ideally quantitatively) assessing the response of an individual horse to a targeted “intervention”, for example not too dissimilar to using flexion tests in order to elicit a change in limb-related lameness.

Changes in back movement have been reported during ridden exercise with camera-based techniques. However, due to marker occlusion by tack (saddle) and rider, the kinematics of the cranial thoracic spine have not been reported [21,22,23]. It seems likely that, since rotational movements are altered caudal to the saddle [21,22,23], the cranial segments (beneath the saddle and rider) will also undergo changes in movement in relation to the added weight of the rider and the presence of the saddle. These movements can now be measured—owing to the miniaturization of inertial measurement units (IMUs)—along the midline of the back, including beneath the saddle [24,25,26].

The aim of this study was to quantify the external kinematics of the thoracolumbosacral area in horses in trot in an unloaded condition (in-hand) compared with when ridden in sitting trot and seated canter with a dynamic load (rider). It is hypothesised that: (1) pitch (differential pitch, rotation around transverse (lateral–lateral) axis) and heading values (differential yaw, rotation around vertical axis) of the caudal thoracic and lumbosacral area will increase when ridden in sitting trot and canter compared with the unloaded condition; (2) differential pitch and heading values will alter in the cranial thoracic region when ridden in sitting trot and canter compared to the unloaded condition without a rider

## 2. Materials and Methods

This study was approved by the Royal Veterinary College ethics and welfare committee, project number URN 20181785-2. Informed, written consent was obtained prior to participation in the study. At the time of the study, all riders were free from any injuries and could withdraw their participation and that of their horses from the study at any point.

### 2.1. Horses

Ten elite dressage horses were used in this study. Eight geldings and two mares, with a mean (±standard deviation) value for height at the withers of 1.70 ± 0.03 m, body mass of 600 ± 24 kg and age 11 ± 1 years were recruited. Horses were housed at two different facilities with data collection taking place at two locations. Horses were all part of an extensive equine sports science and medicine programme including regular therapy and veterinary assessments. On the day preceding data collection all horses were assessed by their respective veterinarian—this assessment included visual observations in walk and trot in a straight line on a firm level surface as well as flexion tests of all four limbs: no lameness was observed. On the day of data collection the horses’ gait asymmetry was quantified using a validated sensor system [27]. In addition, the horses underwent a subjective physiotherapy examination by a chartered veterinary physiotherapist.

### 2.2. Riders

Two male and two female Grand Prix Dressage (FEI ranked) riders who were the horses’ regular riders were recruited with an average (±standard deviation) height 1.82 ± 0.08 m and body mass 74 ± 1 kg. Three horses were ridden by one male rider, three horses were ridden by one female rider, two horses were ridden by one male rider and two horses were ridden by one female rider, each rider rode one horse once.

### 2.3. Saddles, Girths and Bridles

Horses were ridden in their usual saddle, girth and bridle. Static and dynamic saddle fit was assessed independently by five Society of Master Saddlers Qualified Saddle Fitters. Saddle details have been described elsewhere [28]. Seat size remained the same throughout and the stirrup length which the rider was accustomed to was used throughout. A high withered saddle cloth (H: 58 cm withers to base, 54 cm lowest point to base of cloth, W: 63 cm) was positioned beneath the saddle along with a 5-mm thick layer (Prolite half pad). Girth design and features have been described elsewhere [29]. In brief, an anatomically shaped girth not featuring any elastic was used throughout. All horses were ridden in a snaffle bridle with a correctly fitted noseband.

### 2.4. Kinematics—Inertial Measurement Units

**Figure 1:**
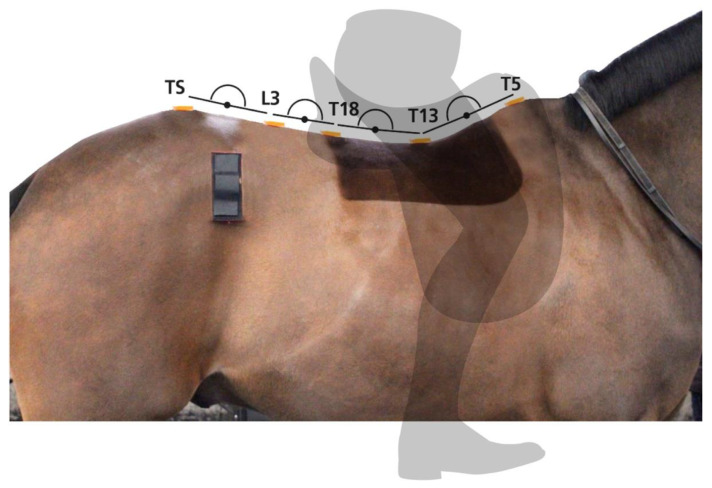
Inertial measurement unit (IMU) sensor locations along the midline of the thoracolumbar region beneath the saddle and rider. Sensors were glued on to the skin over thoracic and lumbar vertebrae (T5, T13, T18, L3). Sensor pouches were used for the sensors attached over the tubera sacrale and left and right tubera coxae.

To measure range of motion, horses were instrumented with eight MTw inertial measurement units (IMU) (Xsens) as part of a sensor-based system (Xsens MTw Awinda, An Enschede, The Netherlands) validated for translational displacements derived from internal tri-axial sensor accelerations rotated into a horse-based reference frame based on the sensor orientation estimate and then double integrated to displacement [27,30]. The IMUs were attached over the poll, withers (T5), vertebral segments of the thirteenth (T13) and eighteenth thoracic vertebrae (T18), the third lumbar vertebra (L3), between the left and right tubera sacrale (TS), and over the left and right tubera coxae. Skin-mounted sensors were attached along the external landmarks of the thoracolumbar spine representing T5, T13, T18, L3 and TS with glue onto the clipped hair using hair extension glue (Salon Pro, London, UK). The remaining sensors (poll and tubera coxae) were attached using custom-made pouches and double-sided tape (Figure 1). The same technician applied all sensors throughout the study. Sensor data were collected at 60 Hz per individual sensor channel [31] and transmitted via a proprietary wireless data transmission protocol (Xsens) to a receiver station (Xsens MTw Awinda, An Enschede, The Netherlands) connected to a laptop computer running MTManager (Xsens, An Enschede, The Netherlands) software. IMU specifications: internal sampling rate 1000 Hz; buffer time up to 30 s; dimensions 47 × 30 × 13 mm; mass 16 g; operating temperature range 0–50 °C; and dynamic accuracy 0.75 degrees root mean square (RMS) (roll/pitch) and 1.5 degrees RMS (heading).

IMU data were processed following published protocols [27]. In brief, tri-axial sensor acceleration data were rotated into a gravity (z: vertical) and horse-based (x: craniocaudal and y: mediolateral) reference frame and numerically double integrated to displacement. Displacement data were segmented into individual strides based on vertical velocity of the sacrum sensor [32], and median values for the following kinematic variables were calculated over all available strides for each exercise condition.

**Figure 2:**
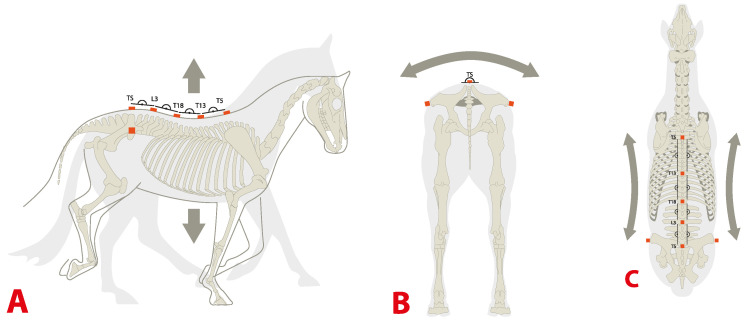
IMU sensor locations in the thoracolumbosacral region and illustration of rotational movement parameters. Differential pitch, roll and heading rotational movements were calculated between adjacent sensors positioned at T5, T13, T18, L3 and TS. Sensor pouches were used for the left and right tubera coxae. (**A**) = pitch, (**B**) = roll and (**C**) = heading.

Orientation–time signals for differential roll, pitch and heading values of T5, T13, T18, L3 and TS were used to calculate differential rotational movements by subtracting signals of adjacent sensors from each other (T5-T13, T13-T18, T18-L3, L3-TS). This method was applied to differential pitch values, differential roll (rotation around longitudinal (craniocaudal) axis) and differential heading values of the upper body landmarks of the thoracolumbar spine and resulted in differential orientation–time signals in degrees in analogy to the method introduced in [33] for flexion–extension (Figure 2).

Outcome parameters for the IMU derived data for the three conditions (in-hand trot, sitting trot and canter) are differential pitch, roll and heading values for T5–T13, T13–T18, T18–L3 and L3–TS.

### 2.5. Study Protocol

#### 2.5.1. In-Hand Trot Data Collection

All horses were walked in-hand (unloaded) with a bridle on and walked in both a clockwise and anticlockwise direction around the arena (20 × 60 m) for twenty minutes. IMUs were fitted to the poll and along the thoracolumbosacral regions and left/right TC as discussed previously. Horses were then trotted in-hand wearing a snaffle bridle, in a straight line in the arena capturing a total of 40 ± 3 strides (6 straight line runs with the horse turning around at each end, the two strides before and after each turn were not included in the analysis). Each horse was trotted in-hand by their respective groom.

#### 2.5.2. Ridden Data Collection

Horses were prepared for ridden exercise with the fitting of the saddle, girth, saddle cloth and half pad as previously described. Care was taken to ensure that the T5, T13 and T18 sensors were not in contact with the medial margins of the saddle panel. One qualified saddle fitter verified sensor location visually and manually by placing their hand beneath the pommel and palpating the lateral edges of the T13 sensor. This observation was made with and without the rider mounted. Each horse underwent a 25-min warm up protocol, self-prescribed by the rider, which included walk, rising/sitting trot and canter on both the left and right reins. Warm up also included lateral work with four half passes, shoulder in and travers (60 m) in trot and canter in both a left and right direction. After the warm-up period had been completed, the kinematics of the thoracolumbar spine were quantified in a straight line in sitting trot and canter, with the rider remaining seated throughout the motion cycle.

**Figure 3:**
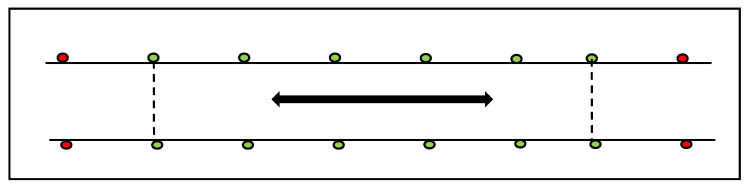
Diagram illustrating the experimental track. The experimental track represented by the green markers and the start and end points being represented by the red markers. The experimental track allowed 11 straight strides in sitting trot and 15 strides in canter to be captured, with both the start and end points being determined by two red cones.

A straight-line experimental track (50 m × 1.5 m) was created in the middle of the arena using spherical cones. The arena dimensions allowed for 11 straight strides in sitting trot and 15 in canter to be captured, with both the start and end points being determined using two cones. All measurements (in-hand and ridden) were performed in an indoor (20 m × 60 m) arena on a wax-coated surface. The surface was groomed prior to, and in between, each horse. Six repeats were captured of the straight-line portion of the track (moving through the experimental track) with the horse approaching from a left (3 repeats) and right (3 repeats) direction in sitting trot and canter (3 repeats left lead and three repeats right lead) (Figure 3). Speed was monitored by the same technician using a stopwatch, with start and end points being defined by two markers that were positioned at the start/end of the experimental track. 

### 2.6. Statistical Analysis

Statistical analysis was performed in SPSS (vers. 26 IBM, Armonk, NY, USA). A general linear mixed model was used for kinematic data with condition (in-hand, sitting trot and canter) and direction (straight line portion of the horse ridden on the left rein or on the right rein through the experimental area) defined as fixed factors and horse defined as a random factor. The significance level throughout was set to *p* ≤ 0.05. A Bonferroni post hoc analysis was carried out to determine pairwise differences between conditions. Instead of applying the Bonferroni correction on the significance level, alpha, this study reported the Bonferroni adjusted *p*-values (*p*-values based on Fisher’s Least Significant Difference (LSD) multiplied by the number of comparisons done). This allows assessment of significance with reference to the traditional alpha of 5%, without increasing type II errors.

## 3. Results

### 3.1. Horse Inclusion

From the subjective veterinary assessment the day preceding the experiment, all horses were deemed fit to perform. From the physiotherapy assessment (on the day) all horses were deemed fit to perform. From the objective movement asymmetry measures, horses had (mean ± SD) asymmetry values (in mm): Poll Min_Diff_ 9.3 ± 8.7, Poll Max_Diff_ −2.0 ± 5.5, Pelvis Min_Diff_, 3.1 ± 2.7, Pelvis Max_Diff_ 2.9 ± 7.6 and Hip Hike Difference (HHD) 4.9 ± 14.3.

### 3.2. Differential Rotational Movement of the Thoracolumbosacral Spine

#### 3.2.1. T5–T13

**Figure 4:**
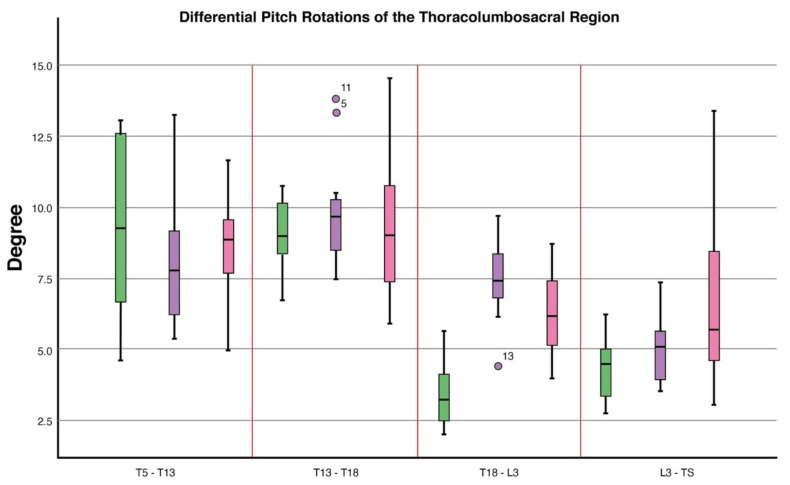
Boxplots displaying differential pitch rotations of the thoracolumbosacral spine in 10 dressage horses whilst trotting in-hand and when being ridden in sitting trot and canter. At T18-L3, differential pitch rotations differed between conditions (*p* = 0.01) where an increase in differential pitch rotations when in sitting trot (*p* ≤ 0.0001) and canter (*p* ≤ 0.0001) was found when compared to trotting in-hand. At L3-TS differential pitch rotations differed between conditions (*p* = 0.005) where an increase in differential pitch rotations was Figure 0. When compared to trotting in-hand. In canter, differential pitch rotations increased when compared to sitting trot (*p* = 0.01). Explanation of symbols: the central line represents the median; the box represents the 25th and 75th percentiles; the whiskers represent the maxima and minima not considered to be outliers; ° represents outliers. Boxplot ID: green = in-hand trot, purple = sitting trot and pink = canter.

**Figure 5:**
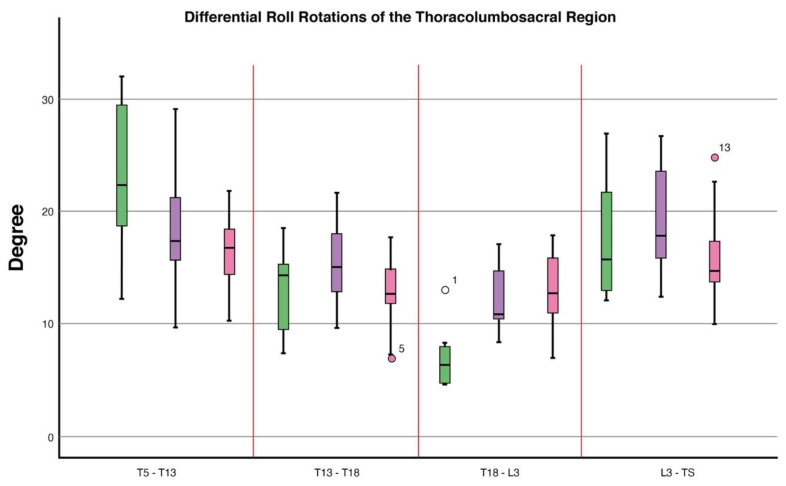
Boxplots displaying differential roll rotations of the thoracolumbosacral spine in 10 dressage horses whilst trotting in-hand and when being ridden in sitting trot and canter. At T5-T13, differential roll rotations differed between conditions (*p* = 0.04) where a decrease in differential roll rotations when in canter compared to trotting in-hand (*p* = 0.005) was found. At L3-TS, differential roll rotations differed between conditions (*p* = 0.05); however, no differences were found between conditions in post hoc analysis. Explanation of symbols: the central line represents the median; the box represents the 25th and 75th percentiles; the whiskers represent the maxima and minima not considered to be outliers; ° represents outliers. Boxplot ID: green = in-hand trot, purple = sitting trot and pink = canter.

**Figure 6:**
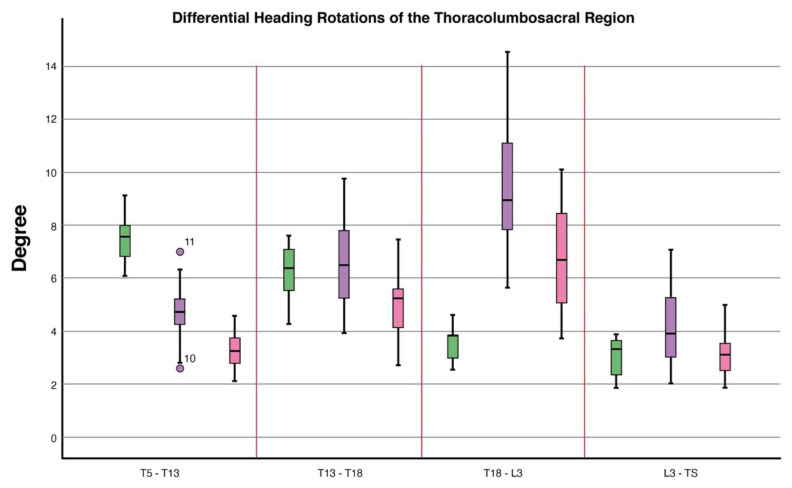
Boxplots displaying differential heading rotations of the thoracolumbosacral spine in 10 dressage horses whilst trotting in-hand and when being ridden in sitting trot and canter. At T5-T13, differential heading rotations differed between conditions (*p* ≤ 0.0001) where a decrease in sitting trot (*p* ≤ 0.0001) and canter (*p* = 0.0001) when compared to trotting in-hand was found. At T18-L3 differential heading rotations differed between conditions (*p* ≤ 0.0001) where an increase in sitting trot (*p* ≤ 0.0001) and canter (*p* = 0.002) when compared to trotting in-hand was found. At L3-TS, differential heading rotations differed between conditions (*p* = 0.01) with an increase when in sitting trot (*p* = 0.02) compared with canter. Explanation of symbols: the central line represents the median; the box represents the 25th and 75th percentiles; the whiskers represent the maxima and minima not considered to be outliers; ° represents outliers. Boxplot ID: green = in-hand trot, purple = sitting trot and pink = canter.

Differential roll values differed between conditions (*p* = 0.04). Post hoc analysis showed a decrease in differential roll values when in canter (Estimated Marginal Mean (EMM) (S.E)): (16.6° (1.4)) compared to trotting in-hand (23.5° (1.7)), (*p* = 0.005). Differential heading values differed between conditions (*p* ≤ 0.0001). Compared to trotting in-hand (7.4° (0.4)), post hoc analysis showed a decrease in differential heading values when in sitting trot (5.1° (0.2), (*p* ≤ 0.0001) and canter (6.5° (0.4)), (*p* = 0.0001) (Table 1, Figure 4, Figure 5 and Figure 6).

#### 3.2.2. T13–T18

Differential heading values differed between conditions (*p* ≤ 0.0001). Post hoc analysis showed an increase in differential heading values when ridden in sitting trot (6.5° (0.4)) compared to trotting in-hand (6.3° (0.5)), (*p* = 0.02). In canter (4.9° (0.4)), differential heading values decreased when compared to sitting trot (6.5° (0.4)), (*p* = 0.0001) (Table 1, Figure 4, Figure 5 and Figure 6).

#### 3.2.3. T18–L3

Differential pitch values differed between conditions (*p* = 0.01). Compared to trotting in-hand (3.4° (0.4)), post hoc analysis showed an increase in differential pitch values when in sitting trot (7.5° (0.3)), (*p* ≤ 0.0001) and in canter (6.3° (0.3)), (*p* ≤ 0.0001). Differential heading values differed between conditions (*p* ≤ 0.0001). Compared to trotting in-hand (3.5° (0.7), post hoc analysis showed an increase in differential heading values when in sitting trot (9.6° (0.4)), (*p* ≤ 0.0001) and in canter (6.7° (0.4)), (*p* = 0.002). Differential heading values were also significantly higher in sitting trot compared to canter (*p* ≤ 0.0001) (Table 1, Figure 4, Figure 5 and Figure 6).

#### 3.2.4. L3–TS

Differential pitch values differed between conditions (*p* = 0.005). Post hoc analysis showed an increase in differential pitch values when in canter (6.5° (0.5)) compared to trotting in-hand (4.3° (0.6)), (*p* = 0.006). In canter, differential pitch values were higher than in sitting trot (4.9° (0.6)), (*p* = 0.01). Differential heading values differed between conditions (*p* = 0.01). Differential heading values were increased in sitting trot (4.0° (0.2)) compared to canter (2.9° (0.3)), (*p* = 0.02). Differential roll values differed between conditions (*p* = 0.05), however, Bonferroni corrected post hoc pairwise comparisons failed to identify any significant differences between conditions (Table 1, Figure 4, Figure 5 and Figure 6).

Directional fixed factor: no differences in differential rotational values were found (trot *p* ≥ 0.07 and canter *p* ≥ 0.20), for any of the thoracolumbosacral regions when the horses entered the experimental track from either the left or right direction.

## 4. Discussion

In this study IMUs were positioned on the skin surface overlaying bony structures of the dorsal spinous processes of the thoracolumbosacral spine and differential rotational movements were quantified.

IMUs have been used extensively for quantifying axial kinematics for over ground locomotion in straight lines [34,35,36] and when circling [34,37,38,39] in lame and non-lame horses [37,40,41,42]. Their outputs have been validated against optical motion capture for quantifying translational movements of the upper body [27,30] and a direct validation of flexion–extension angles from IMU data has reported differences of <1° [33]. There is, however, at present, no direct validation data available for the quantification of IMU-based thoraco–lumbo–sacral angles compared to motion capture for the two remaining rotational movements (lateral bending and axial rotation). The validation data of IMU-derived translational movements, in two dimensions (mediolateral and dorsoventral) [30], provide some indication that the rotational IMU estimates are adequate representations in comparison to motion capture, since the integration process that is required to calculate translational displacement in the world- (or horse-) based reference frame from tri-axial accelerations sensed in the sensor-based reference frame relies intrinsically on an accurate orientation estimate at each timepoint [27]. Further work is required to quantify IMU based angles, particularly for lateral bending, as lateral bending and lateral excursion do not follow a similar pattern [3]. In horses trotting on a treadmill with skin-mounted markers, quantifying kinematics of the thoracolumbar region, lateral bending was greatest at T10 and decreased caudally to L1, whereas lateral excursion was greatest at T17 and lowest at T10 [3].

It has been reported that rotations derived from skin-mounted IMUs differ considerably from the internal vertebral rotations (in particular for axial rotation of the pelvis) [43], which should not come as a big surprise based on previous studies reporting considerable skin displacement compared to bony landmarks [14,15]. In this manuscript, in order to delineate our external measurements based on skin-mounted IMUs from the previously conducted measurements of the underlying bony landmarks characterizing axial rotation, flexion–extension and lateral bending of the spine [6,7,8], we are using the terms roll, pitch and heading.

A previous study, conducted with a predecessor version of the sensors used here, aimed at establishing how well the roll angle (related to axial rotation) of a skin-mounted IMU placed over the midline of the pelvis (over the sacrum) would be able to estimate tuber coxae movement quantified from skin-mounted IMUs [44]. Tuber coxae movement is generally considered an important visual parameter for decision making about movement asymmetries in hindlimb lame horses [45]. Our previous study [44] is in agreement with Goff et al. [43] which indicated that, across horses, the midline rotation measured from a skin-mounted IMU does not result in accurate estimates of tuber coxae movement. However, the results of our previous study [44] also indicate that changes between movement conditions within a subject, specifically between straight line and lungeing and before/after flexion tests can be quantified with adequate accuracy (between 1 mm and 6 mm difference between measured and estimated tuber coxae movement asymmetry). This provides practically relevant data supporting the use of skin-mounted IMUs for investigating kinematic changes of external landmarks between conditions, which is the approach of the current study quantifying differences in each horse between different exercise conditions. Visual assessment of external landmarks is an essential part of the veterinary lameness examination [46]. Established quantitative camera- and sensor-based methods can accurately and precisely measure the movement of these external landmarks [32,35,36,38,39,47,48,49,50,51,52] and now provide evidence underpinning veterinary decision making, for example quantifying the effects of diagnostic analgesia [36,38,53,54,55] and compensatory movements [34,39,56]. It has been shown that thoracolumbosacral range of motion is altered after eliminating pain-causing lameness and quantitative measurement of back movement may further inform veterinary decision making [37,41] even if the measurements do not exactly represent the movement of the underlying bony structures [43]. Whether the IMU derived movement parameters of the thoracolumbosacral area can make useful contributions to veterinary decision making deserves further attention.

In trot, the differential pitch values presented here differ from flexion–extension angles derived from motion capture with bone-fixated [6] and skin-mounted markers [3,5,13,42,57]. With bone-fixated markers, flexion–extension of the vertebra ranged from 2.8° ± 0.8 to 4.9° ± 1.4 with the greatest flexion–extension occurring at the 10th thoracic vertebra [6]. In the current study, when trotting in-hand, the differential pitch values ranged from 9.2° (0.8) to 4.3° (0.6) with the greatest pitch angle occurring in the cranial thoracic region (T5-T13). The differences between motion capture angles and sensor-based angles generally lie in the cranial thoracic region. These differences could be explained by the skin-mounted IMU sensor of the withers being furthest away from the vertebral body in comparison to the more caudal sensor locations. The differential pitch values for the caudal thoracic and lumbar regions appear to be similar to flexion–extension angles obtained from bone-fixated [6] and skin-mounted markers [3,5,13,21,42,57].

In accordance with our experimental hypothesis, compared to the unloaded condition (trot in-hand) the results of this study indicate that differential pitch values of each segment differ between conditions: sitting trot and seated canter. In sitting trot compared to trotting in-hand (unloaded), the caudal thoracic–lumbar region (T18-L3) showed increased pitch rotations. In horses trotting on a treadmill with a saddle and lead weight (75 kg), the lumbar spine has been shown to extend whilst overall range of motion remained the same [21]. In the current study we express values as dynamic pitch rotations and do not attempt to differentiate between flexion and extension. The weight of the riders in the current study was 74 ± 1 kg and the values being presented here (sensor-based angles) are in the region of those presented for skin marker angles when considering the overall range of motion of the third and fifth vertebrae of the lumbar spine [21] (≤1.5°) compared with the region of L3–TS.

Compared to in-hand trot and sitting trot, increased differential pitch values were found when in canter for the caudal thoracic and lumbosacral regions (T18–L3, L3–TS). During ridden exercise the increase in pitch values in sitting trot and canter of the caudal thoracic and lumbosacral spine seems reasonable to expect given the linkage between the pelvis and spine. Differences in pitch values of the back segments appear to be influenced by gait, which may be explained by differences in limb rotations relative to the body. In trot the hindlimb rotates from the hip, whereas in the canter the limbs have a pendular movement originating from the lumbosacral region. These movements are facilitated by activation of the sub lumbar, intrinsic and extrinsic musculature of the hindlimb and the lumbodorsal fascia stretching and recoiling elastically aiding force generation. The force generation and transmission, which are likely to influence kinematics of the vertebral column which is being reflected in the upper body skin-mounted sensors.

Due to marker occlusion of the cranial thoracic spine, studies which have reported kinematics of the back with a saddle + rider (or weight) have been limited to the kinematics caudal to the saddle [21,22]. Using validated IMUs and published methods [27,30,33], IMUs which are positioned beneath the saddle provide a means of quantifying thoracolumbar kinematics. In a study of three horses, comparing the kinematics of the thoracolumbar spine when trotting in-hand compared with rising trot, the authors presented values for both the seated and standing components of rising trot [26]. In the seated phase, the part of the back under the seat of the rider was less mobile with decreased flexion–extension in the mid thoracic (T12–T16) and lumbar region (T16–L2) [26], whilst the cranial thoracic spine (T6–T12) showed an increase in overall flexion–extension values. The findings from Martin et al. (2017) differ from the findings of the current study, where no change in pitch rotations of the cranial thoracic spine was found between trotting in-hand (unloaded) and sitting trot (ridden); therefore, we partially refute our second hypothesis. When interpreting these differences, it is important to note that the previous study used jumping horses (Personal Communications P. Martin) which may have different conformations [58] to the horses used in the current study. Furthermore, we quantified back movement using dressage saddles whereas the aforementioned study used jumping saddles [26]. Lastly, we made comparisons with the “seated” phase of the trot cycle (and canter), with the assumption that back movement would follow similar amplitudes and movement distribution between the different rotational components for each diagonal stance phase, (i.e., rotational back movement would be similar during the ground contact of both diagonal pairs of limbs). However, rising trot and sitting trot induce different dynamic forces on the locomotor apparatus [20,59,60] and it seems likely that vertebral movement and amplitudes for vertebral segments vary between these two different seating positions. Therefore, future work should quantify back movement under various riding positions: sitting and rising trot and two-point position allowing for a more comprehensive biomechanical interpretation of back movement.

Similar to pitch values, our heading rotations in trot differ from motion capture angles for bone-fixated [6] markers. Lateral bending of the thoracolumbar spine with bone-fixated markers ranged from 4.9° ± 1.2 to 3.6° ± 1.8 with the greatest lateral bending occurring at the sixth thoracic vertebra [6]. In the current study, when trotting in-hand (unloaded), differential heading values ranged from 7.4° (0.4) to 3.0° (0.4) with the greatest heading angle occurring in the cranial thoracic region (T5-T13). Similar to the differential pitch values, our sensor-based values are higher than bone-fixated markers, with the differences generally in the cranial thoracic region. Similar to the differential pitch values, the heading values for the caudal thoracic and lumbar regions seem similar to those presented for bone-fixated markers [6]. When comparing skin-mounted marker-based studies, our heading values are in the region of lateral bending angles reported for asymptomatic horses trotting on a treadmill [42] and horses trotting on a treadmill from two different laboratories [13]. In horses who are deemed fully functioning, our values are similar for the cranial thoracic spine but are less for the caudal lumbar spine; the reason for this is unknown and warrants further investigation. One explanation could be that in the current study the horses were all elite dressage horses, of a similar type and conformation, compared to the Johnson et al. (2002) study where the horses were a mixture of dressage, event and jumping [58].

In accordance with the second experimental hypothesis, the results of our study show a decrease in heading values in the region of the thoracic spine (T5–T13), when ridden in sitting trot and canter, compared to the in-hand condition (unloaded). The fact that there are no studies reporting lateral bending or heading values for the cranial thoracic spine under ridden conditions limits possible comparisons. It is speculated that this decrease in movement amplitude may be indicative of an attempted “stiffening” mechanism, in order to better withstand the dynamic forces of the rider [59] (and saddle) and more efficiently transmit dynamic forces from the forelimb (and head and neck) to the cranial region of the thoracic spine; this idea may warrant further investigation.

The horses in this study were assessed for upper body movement symmetry quantitatively and were assessed visually for lameness by a veterinarian. All horses were deemed non-lame by visual assessment. Average movement symmetry values across horses were generally small and with the exception of Poll MinDiff (value of 9 mm) were within the thresholds of 8 mm for head movement symmetry and 4 mm for pelvic movement symmetry (thresholds presented in McCracken et al., 2012 [61]) adapted with equations presented in Pfau et al., 2016 [49]. It should be emphasized that the clinically applied thresholds are chosen to provide a high sensitivity at the cost of a lower specificity. This is appropriate for the clinical lameness examination where the task is to identify the affected limb(s). A higher threshold value of 14.5 mm for Poll MinDiff has on the other hand been suggested based on a comparison of visual and quantitative data in Thoroughbred racehorses [62] which is similar to the daily and weekly variation of 14 mm and 19 mm, respectively, for this parameter in Thoroughbred racehorses in training [63]. This should be taken into account when interpreting movement asymmetry values in a ”screening” scenario.

Lastly, this study quantified differential rotations of the thoracolumbar spine in horses who were trotted in-hand and then ridden in sitting trot and canter in order to quantify changes in back movement with two specific types of ridden exercise (sitting trot and canter). Future research, quantifying rotational movement of the thoracolumbosacral region when ridden in different riding positions (rising trot, two-point, correct/incorrect diagonal) [20], at different gaits, and with varying head and neck positions [64,65] is warranted.

## 5. Conclusions

Using skin-mounted IMUs, this study has reported changes in external upper body landmarks of the thoracolumbar area when horses are trotting in a straight line and ridden in sitting trot and canter. With ridden exercise, differential heading values decreased in the cranial thoracic region and increased in the caudal thoracic/lumbosacral region. Differential pitch values increased in the caudal thoracic/lumbosacral region whilst the cranial thoracic region appears to remain unaffected by ridden exercise. The method presented here provides quantitative data from external upper body landmarks of the thoracolumbosacral area in horses during in-hand and ridden exercise. It should be further investigated whether such quantitative data can make a useful contribution to veterinary decision making in the context of the management of back related conditions.

## Figures and Tables

**Table 1:** Display of the estimated marginal mean (EMM) ± standard error (SE) for differential pitch, roll and heading rotational values for the thoracolumbosacral spine in the unloaded condition (in-hand (IH)), and ridden conditions (collected trotted trot (CT) and collected canter (CC)) from 33 straight trot strides and 45 straight canter strides. Table showing gait effect and outcome of Bonferroni post hoc tests (*p* ≤ 0.05).

	Segment	In-Hand (Unloaded) Trot Straight EMM SE (±)	Sitting Trot Pooled EMM SE (±)	Canter Pooled EMM SE (±)	Gait Effect *p* Value	Bonferroni Post Hoc
Differential Pitch Rotation	T5-T13 (°)	9.2 (0.8)	8.3 (0.8)	8.7 (0.6)	0.23	-
	T13-T18 (°)	9.0 (0.7)	10.2 (0.5)	9.4 (0.5)	0.27	-
	T18-L3 (°)	3.4 (0.4)	7.5 (0.3)	6.3 (0.3)	0.01	IH < CT, *p* < 0.0001 IH < CC, *p* < 0.0001 CT > CC, *p* = 0.05
	L3-TS (°)	4.3 (0.6)	4.9 (0.6)	6.5 (0.5)	0.005	IH < CC, *p* = 0.006 CT < CC, *p* = 0.01
Differential Roll Rotation	T5-T13 (°)	23.5 (1.7)	18.7 (1.3)	16.6 (1.4)	0.04	CT < IH, *p* = 0.09 CC < IH, *p* = 0.005
	T13-T18 (°)	13.2 (1.2)	15.3 (0.9)	13.2 (0.9)	0.07	-
	T18-L3 (°)	7.1 (1.0)	11.9 (0.8)	12.8 (0.8)	0.24	-
	L3-TS (°)	17.4 (1.5)	18.7 (1.5)	16.9 (1.5)	0.05	-
Differential Heading Rotation	T5-T13 (°)	7.4 (0.4)	5.1 (0.2)	3.2 (0.2)	<0.0001	IH > CT, *p* ≤ 0.0001 IH > CC, *p* ≤ 0.0001
	T13-T18 (°)	6.3 (0.5)	6.4 (0.4)	4.9 (0.4)	<0.0001	IH > CC, *p* = 0.02 CT > CC, *p* = 0.001
	T18-L3 (°)	3.5 (0.7)	9.6 (0.4)	6.7 (0.4)	<0.0001	IH < CT, *p* ≤ 0.0001 IH < CC, *p* = 0.002 CT > CC, *p* ≤ 0.0001
	L3-TS (°)	3.0 (0.4)	4.0 (0.2)	2.9 (0.3)	0.01	CT > CC, *p* = 0.02

## Data Availability

The data presented in this study are available on request from the corresponding author. The data are not publicly available due to confidentiality.
